# Heparin-calibrated anti-factor Xa activity as a surrogate marker for direct oral anticoagulant levels in acute stroke care: a large observational single-center study

**DOI:** 10.1186/s42466-026-00524-1

**Published:** 2026-08-18

**Authors:** Martin Büchsel, Katharina Lisko, Hanna Gölz, Jürgen Bardutzky, Heinz Wiendl, Saúl Beltrán Felipa, Johann Lambeck

**Affiliations:** 1 Institute for Clinical Chemistry and Laboratory Medicine, Faculty of Medicine, Medical Center - University of Freiburg, University of Freiburg, Hugstetter Str. 55, 79106 Freiburg, Germany; 2 Department of Neurology and Clinical Neurophysiology, University of Freiburg Medical Center, Breisacherstr. 64, 79106 Freiburg, Germany

**Keywords:** DOAC, Anti-factor Xa activity, Thrombolysis, Stroke, Anticoagulation

## Abstract

**Background and Purpose:**

Direct oral anticoagulants (DOAC) complicate therapeutic decision-making, as rapid assessment of anticoagulant activity is critical in emergency patients, particularly in acute ischemic stroke (AIS). The administration of systemic thrombolysis (IVT) in anti-coagulated patients requires careful risk–benefit evaluation, given the potential for hemorrhagic complications. Substance-specific DOAC plasma level measurements are often unavailable outside tertiary centers. This study evaluated whether widely available heparin-calibrated anti-factor Xa (anti-Xa) activity can reliably estimate clinically relevant DOAC levels and support urgent clinical decision-making.

**Methods:**

This retrospective single-center study (2015–2023) analyzed 969 patients treated with apixaban, rivaroxaban, or edoxaban. All patients had parallel measurements of anti-Xa activity and specific DOAC levels. Correlations were assessed using Spearman’s coefficient. Receiver operating characteristic (ROC) analyses were performed to derive anti-Xa cut-off values corresponding to DOAC concentrations of 30, 50, and 100 ng/mL.

**Results:**

Anti-Xa activity strongly correlated with DOAC plasma levels for all substances (Spearman’s ρ ≈ 0.95–0.96). ROC analyses demonstrated excellent diagnostic accuracy, with areas under the curve ≥ 0.97 for all evaluated thresholds. Similar anti-Xa cut-offs were observed for apixaban and rivaroxaban, whereas these were lower for edoxaban. For all DOACs combined, anti-Xa cut-offs of approximately 0.36U/mL, 0.56U/mL, and 1.19U/mL corresponded to DOAC levels of 30, 50, and 100ng/mL, respectively, with high sensitivity and specificity.

**Conclusions:**

Heparin-calibrated anti-Xa activity is a reliable surrogate marker for clinically relevant DOAC levels. Defined anti-Xa cut-offs are highly specific and sensitive and enable rapid and standardized assessment of anticoagulant activity in this cohort.

## Introduction

Since their introduction in 2008, the use of direct oral anticoagulants (DOACs) has fundamentally changed the management of thromboembolic diseases. DOACs offer two main advantages over classic vitamin K antagonists; namely, (i) a superior risk-benefit profile, particularly in relation to intracranial bleeding, and (ii) a lack of necessity for regular laboratory checks [1]. These advantages have therefore led to a rapid rise in the use of DOACs in patients with atrial fibrillation, deep vein thrombosis or pulmonary artery embolism.

However, despite their widespread application, DOACs are associated with particular challenges in acute medicine. For example, in time-critical situations, significant bleeding or the need for urgent surgical intervention, treating physicians must decide on appropriate therapeutic measures within a very short time frame, and any uncertainty regarding the extent of anticoagulation is potentially harmful.

Although substance-specific laboratory analyses exist to determine DOAC levels, such services are unavailable in non-specialised centres (i.e. tertiary care) such as smaller hospitals, which means that treatment decisions often have to be made without reliable laboratory values, or are based on mere dipstick testing with reduced accuracy [2]. Alternatively, patients have to be transferred to a specialised centre, thus resulting in the loss of valuable time.

In this context, it is important to distinguish between the different methods used to quantify factor Xa inhibitor levels. DOAC concentrations can be determined either by liquid chromatography–tandem mass spectrometry or by drug-specific chromogenic anti-factor Xa assays calibrated for the respective factor Xa inhibitor. In the present study anti-factor Xa activity was measured calibrated for apixaban, rivaroxaban, or edoxaban, with the results reported as anti-factor Xa -derived DOAC concentrations in ng/mL. In parallel, anti-factor Xa activity was measured separately using a heparin-calibrated assay reported in U/mL. Thus, the study compared two separately obtained anti-factor Xa-based measurements: DOAC-specific assay-derived concentrations referred to as substance-specific levels and heparin-calibrated anti-factor Xa activity.

The clinical relevance is particularly evident in patients with acute ischaemic stroke (AIS), in whom physicians must balance the potential benefit of urgent intravenous thrombolysis (IVT) against a potentially increased risk of bleeding in the presence of residual DOAC activity.

The European Stroke Organisation (ESO) currently recommends the following threshold values for safe application of thrombolysis therapy: <30 ng/ml for DOAC-specific levels [3] or < 0.5 U/ml for heparin-calibrated anti-factor Xa activity [4]. Other experts deviate from these suggestions [5–8], and a recent multicentre retrospective analysis did not identify an increased rate of symptomatic intracranial haemorrhage with DOAC-associated IVT at all [5, 9, 10]. Taken together, these findings highlight the heterogeneity of clinical practice and the urgent need for practical, reliable and rapidly available laboratory parameters for the evaluation of coagulation status in emergency situations.

The present study therefore set out to determine whether (i) the heparin-calibrated anti-factor Xa activity, which is rapidly ascertained in many laboratories, is suitable for reliably estimating the extent of the DOAC effect and (ii) there is a correlation between the heparin-calibrated anti-factor Xa activity and substance-specific levels, and whether a standardised threshold value can be derived from this - even if the exact DOAC taken was unknown.

The development of a standardised coagulation diagnostic approach in DOAC treated patients with AIS based on specific threshold values is a long-term goal and we hope that the findings of this study may further add to the body of knowledge concerning this topic.

## Materials and methods

### Study design

This observational, retrospective single center study analysed laboratory and clinical data from 1187 patients between 2015 and 2023. The study was approved by the Institutional Ethics Committee (24-1081-S1-retro). All clinical data were collected retrospectively. Data are published in aggregated form precluding any conclusions about individual patients and thereby protecting patients’ right to data privacy. The study is registered at the “Freiburg Registry of Clinical Trials” (FRKS) with the ID-number FRKS004985 (registration date: 22.11.2023).

For the initial analysis, all laboratory studies (including parallel DOAC and heparin-calibrated anti-factor Xa activity measurements) that were carried out at the Medical Centre - University of Freiburg were retrieved from patient records. Most cases involved patients admitted to the emergency department of the university hospital with suspected stroke (focal neurological deficits and/or reduced vigilance). An assessement of the anticoagulation parameters including DOAC levels and heparin-calibrated anti-Xa-activity is routine for this cohort. Further parameters included: (i) other factors influencing the coagulation pathway, such as blood counting (especially platelet levels), (ii) aPTT and INR values, (iii) renal function and (iv) intake of other anticoagulants. Moreover, patient characteristics (age, gender), medication, underlying disease warranting DOAC treatment, suspected diagnosis justifying DOAC or heparin-calibrated anti-factor Xa activity measurement, and final diagnosis were also acquired. Patients which were later identified as not having used an oral anti-factor Xa inhibitor (apixaban, rivaroxaban and edoxaban), as well as patients who were on an unspecified oral anti-factor Xa inhibitor were excluded from further analysis.

### Heparin-calibrated anti-factor Xa activity assay and quantification of DOAC-level

We retrospectively correlated heparin-calibrated anti-Xa activity data with substance-specific DOAC levels that had been measured simultaneously. Measurements of heparin-calibrated anti-Xa activity were performed using chromogenic substrate assay (Hyphen Biophen^®^ LRT). For substance-specific DOAC levels anti-factor Xa activity was measured using an chromogenic substrate assay (Hyphen Biophen^®^ LRT) with substance specific calibrators (Hyphen biomedical). All assays were performed on Sysmex CS5100 analyzers.

### Statistical analysis

Statistical analysis was performed using R software (Version 4.5.1). Receiver operating characteristic (ROC) analyses were performed to identify heparin-calibrated anti-factor Xa activity cut-off values for clinically significant DOAC concentrations: Early analysis and guidelines propose threshold values of < 20ng/mL [7], < 30 ng/mL [3] and < 50ng/mL [5] for DOAC-specific levels before IVT in AIS. A value of > 100ng/mL has been widely regarded as unsafe for IVT. Thus, we determined anti-Xa cut-off values at 30 ng/mL, 50ng/mL and 100 ng/mL DOAC-concentrations.

## Results

### Study population

**Fig. 1:**
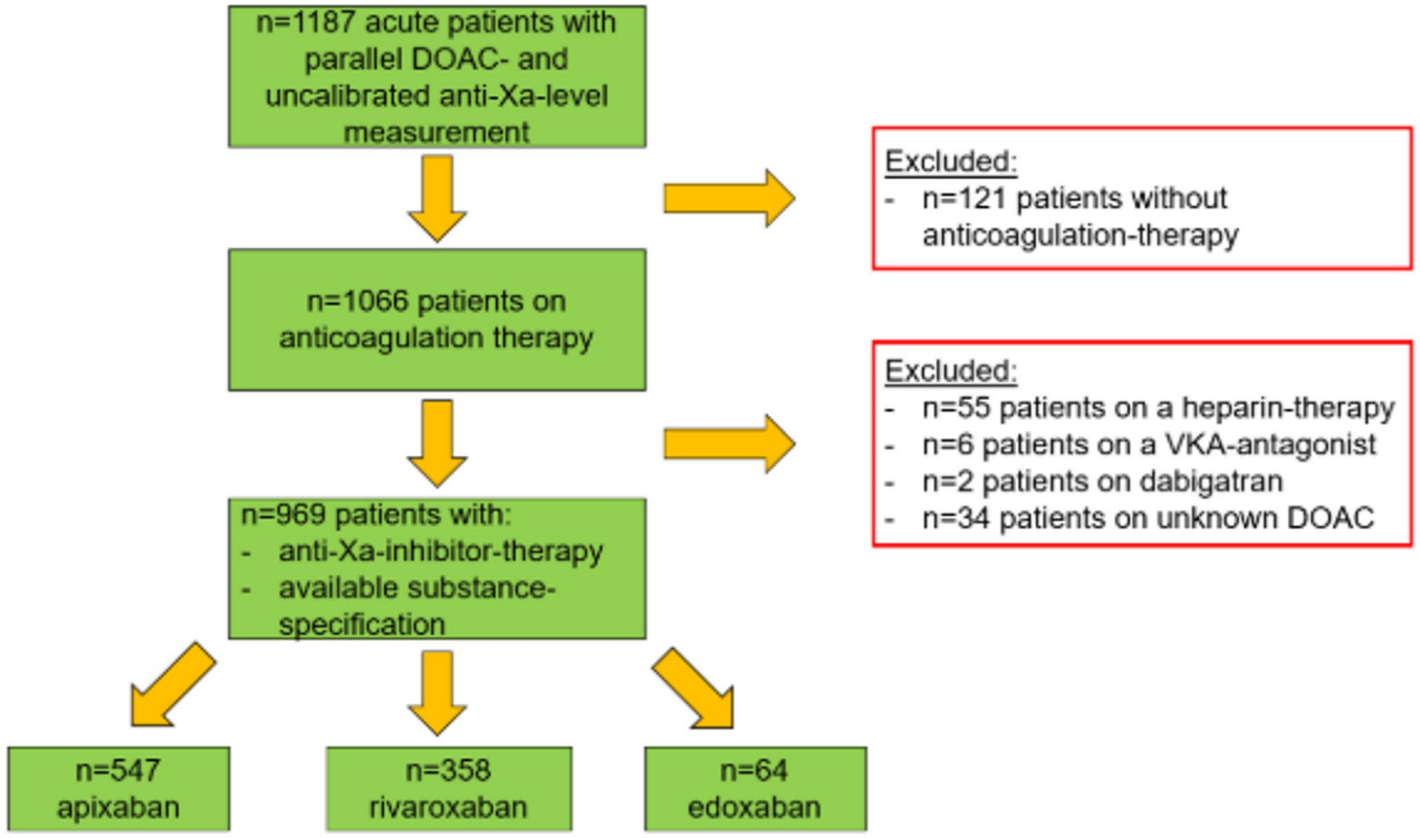
Patient inclusion scheme, all patients (*n* = 1187), DOAC: direct oral anticoagulant, VKA: vitamin K antagonist. Created with Microsoft PowerPoint (Office 2019)

A total of 1187 patients with suspected DOAC intake were screened for eligibility. Of these, 219 subjects did not fulfill the required criteria (anti-Xa therapy, DOAC specification) and were subsequently excluded from further analysis (Fig. 1).

Therefore, 969 subjects were included, with 429 women (44%), and a mean age of 81 years (Table 1).

**Table 1:** Patient characteristics

Parameter	Value
Patients	969
Age (y, IQR)	81 (75–86)
Female, n (%)	429 (44.2)
Reason for anti-Xa measurement, n (%)	
Suspected Stroke	837 (86.4)
Epileptic seizure	77 (7.9)
Trauma	21 (2.2)
Other	34 (2.7)
Final Diagnosis, n (%)	
Ischemic Stroke	429 (44.3)
Intracerebral hemorrhage	153 (15.8)
Other	387 (39.9)
Anticoagulation, n (%)	
Apixaban:	547 (56.4)
Rivaroxaban:	358 (36.9)
Edoxaban:	64 (6.4)
Indication for Anticoagulation, n (%)	
Atrial fibrillation	819 (84.5)
Venous thromboembolism	85 (8.8)
Other indications	65 (6.7)
Creatinine (mg/dl, IQR)	1.06 (0.85–1.34)
eGFR, n (%)	
≥50 ml/min	642 (66.3)
30–49 ml/min	218 (22.5)
< 30 ml/min	100 (10.3)
Anticoagulation paused, n (%)	
No	952 (98.2)
Yes	17 (1.8)

The predominant reason for anticoagulation was non-valvular atrial fibrillation (NVAF; 85%), followed by venous thromboembolism (9%). Apixaban treatment was reported in 547 (56%), rivaroxaban in 358 (37%) and edoxaban in 64 (7%) subjects. 837 patients (86%) were admitted with suspected stroke (focal neurological deficit and/or reduced vigilance), followed by 77 patients with suspected or confirmed epileptic seizure (8%). Other causes included traumatic brain injury, dizziness and a planned intervention such as lumbar puncture or electromyography. AIS was confirmed in 429 patients (44%), and IVT was administered in 36 patients. Intracranial hemorrhage was the second most common diagnosis with 153 patients reported (16%). Other common diagnoses included epileptic seizures, intoxication, delirium and exsiccosis.

Only 18 of the 969 patients (2%) had paused anti-coagulation treatment prior to the onset of neurological symptoms. In 11 cases, the exact duration of this pause was unknown. Notably, 318 patients (33%) exhibited an estimated glomerular filtration rate (eGFR) below 50 ml/min, potentially compromising the pharmacokinetic predictability of DOAC plasma concentrations. DOAC plasma levels below the clinically significant thresholds of 50 ng/ml and 100 ng/mL were identified in 145 (15%) and 378 (39%) patients, respectively.

### Correlation studies and cut-off values for heparin-calibrated anti-factor Xa activity

**Fig. 2:**
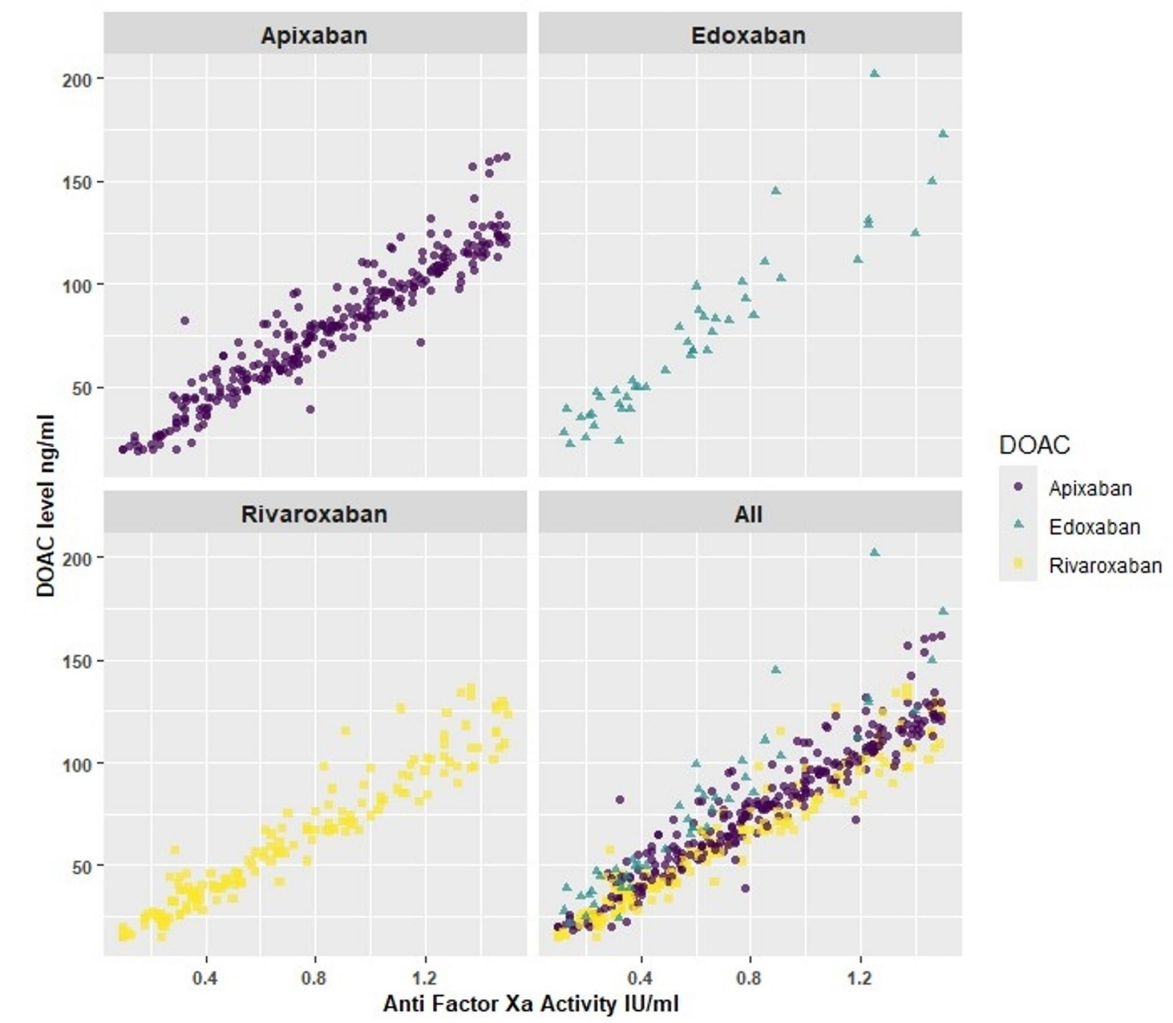
Correlation between heparin calibrated anti-Xa assay and DOAC levels (measured by substance specific calibrated anti-Xa assay). Spearman correlation coefficient was 0.96 (95% CI 0.95–0.96) for apixaban, 0.96 (95% CI 0.93–0.96) for edoxaban, 0.96 (95% CI 0.95–0.96) for rivaroxaban and 0.95 (95% CI 0.94–0.95) for all 3 substances. Created with R software (Version 4.5.1)

Figure 2 shows the correlation between heparin-calibrated anti-Xa activity and DOAC plasma levels. The Spearman correlation coefficient was 0.96 (95% CI 0.95–0.96) for apixaban, 0.96 (95% CI 0.93–0.96) for edoxaban, 0.96 (95% CI 0.95–0.96) for rivaroxaban and 0.95 (95% CI 0.94–0.95) for all 3 substances

**Fig. 3:**
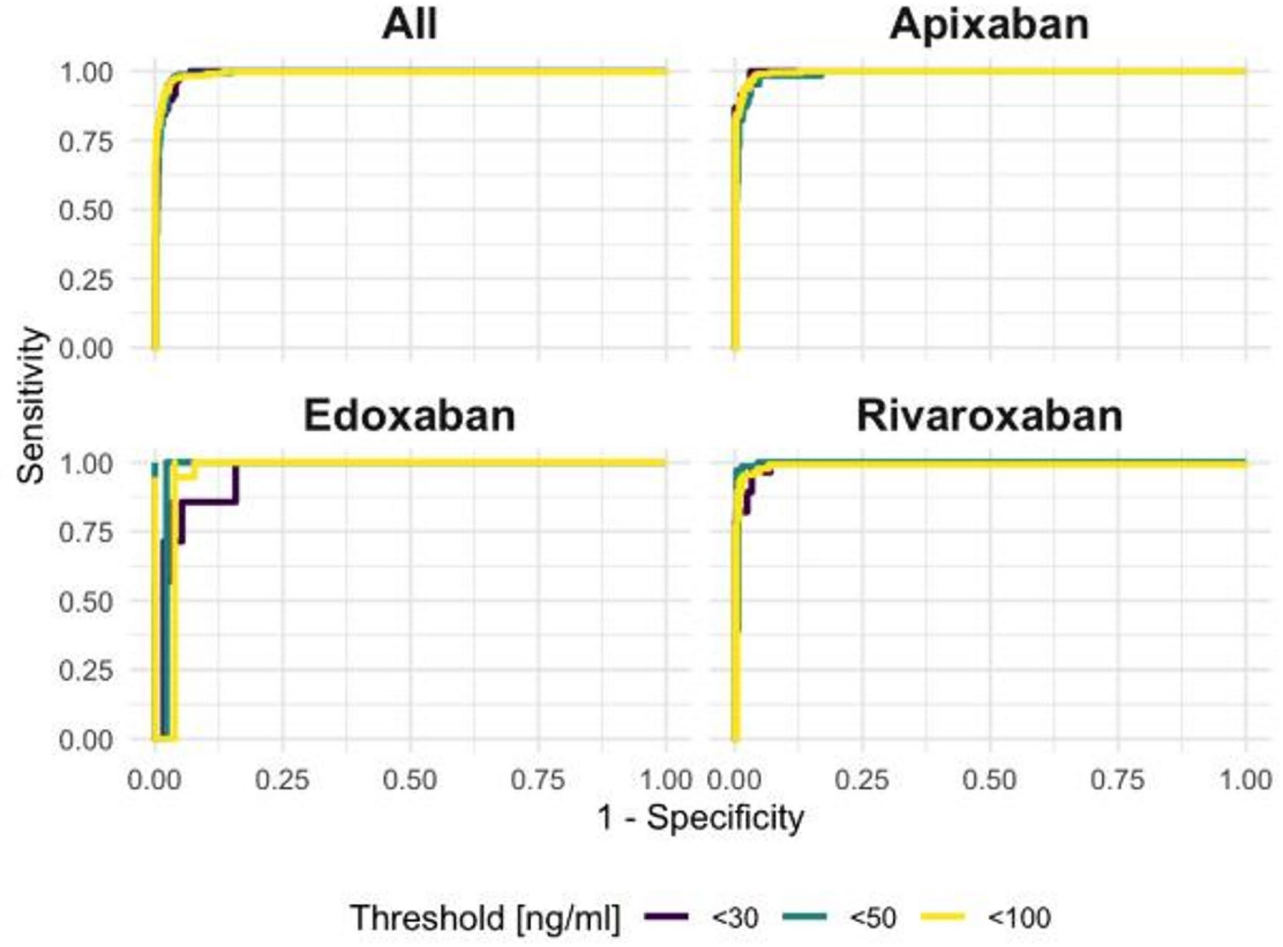
Receiver-operating characteristic (ROC) at clinically relevant thresholds of DOACs. Heparin calibrated anti-Xa assay and DOAC levels were measured in *n* = 969 samples (547 from patients on apixaban, 358 on rivaroxaban and 64 on edoxaban). Created with R software (Version 4.5.1)

Receiver operating characteristic (ROC) analyses were performed to derive anti-Xa cut-off values corresponding to DOAC concentrations of 30, 50, and 100 ng/ml. While thresholds for apixaban and rivaroxaban were comparable, distinctly lower cut-offs were identified for edoxaban (Fig. 3).

For apixaban, the anti-Xa cut-offs of approximately 0.36 U/ml, 0.56 U/ml, and 1.17 U/ml corresponded to DOAC levels of 30, 50, and 100 ng/ml, respectively; for rivaroxaban, the anti-Xa cut-offs of approximately 0.34 U/ml, 0.60 U/ml, and 1.22 U/ml corresponded to DOAC levels of 30, 50, and 100 ng/ml, respectively; while for edoxaban, the anti-Xa cut-offs of approximately 0.33 U/ml, 0.37 U/ml, and 0.83 U/ml corresponded to DOAC levels of 30, 50, and 100 ng/ml, respectively. ROC analyses demonstrated excellent diagnostic accuracy, with areas under the curve ≥ 0.97 for all evaluated thresholds (Table 2).

**Table 2:** ROC (Receiver Operating Characteristic) curve analysis

DOAC	Threshhold	Cut-Off	AUC	AUC_CI	Sens	Sens_CI	Spec	Spec_CI
Apixaban	30	0.355	0.997	0.993–1.000	1.000	0.826–1.000	0.970	0.954–1.000
Apixaban	50	0.560	0.992	0.985–0.998	0.984	0.903–1.000	0.953	0.811–0.975
Apixaban	100	1.165	0.996	0.994–0.999	0.986	0.938–1.000	0.961	0.869–0.982
Rivaroxaban	30	0.335	0.994	0.987–1.000	0.964	0.781–1.000	0.967	0.915–0.994
Rivaroxaban	50	0.595	0.997	0.993–1.000	0.986	0.937–1.000	0.986	0.937–1.000
Rivaroxaban	100	1.215	0.993	0.984–1.000	0.955	0.894–0.985	0.982	0.929–1.000
Edoxaban	30	0.325	0.971	0.926–1.000	1.000	0.714–1.000	0.842	0.754–1.000
Edoxaban	50	0.365	1.000	1.000–1.000	1.000	1.000–1.000	1.000	1.000–1.000
Edoxaban	100	0.830	0.998	0.993–1.000	1.000	0.868–1.000	0.962	0.885–1.000
All	30	0.355	0.992	0.987–0.997	0.983	0.896–1.000	0.954	0.920–0.969
All	50	0.555	0.993	0.989–0.997	0.980	0.933–1.000	0.963	0.891–0.980
All	100	1.185	0.993	0.989–0.997	0.974	0.933–0.990	0.962	0.889–0.980

## Discussion

The presented cohort consists mainly of patients admitted with the suspected diagnosis of acute stroke. A potential DOAC intake can be difficult to clarify in the acute setting. Therefore, we determined the specific DOAC-level and heparin-calibrated anti-Xa activity in parallel in all patients. Patients presenting with acute, disabling ischemic stroke while undergoing direct DOAC therapy may benefit from intravenous IVT, particularly when they do not qualify for mechanical thrombectomy. However, IVT administration in anti-coagulated patients requires careful risk–benefit evaluation, given the potential for hemorrhagic complications. Current guidelines recommend considering IVT in selected patients, based on the time since the last DOAC intake, renal function, and coagulation test results [1, 7]. Nevertheless, no universally accepted threshold exists to safely carry out IVT in this cohort.

Routine determination of plasma DOAC concentrations remains unavailable in many institutions. Prior studies have shown that implementing the testing of DOAC levels can significantly increase the proportion of eligible patients receiving IVT [11]. Given the limited access to substance-specific assays, there is growing interest in utilizing the more widely available heparin-calibrated anti-Xa activity assays as a surrogate marker for DOAC effects [12–14]. The primary objectives of this study were to evaluate whether the heparin-calibrated anti-factor Xa activity is suitable for estimating DOAC-levels, to assess a correlation between these coagulation parameters and finally to establish threshold values for different DOAC-levels (< 30 mg/mL, < 50 ng/mL and 100 ng/mL) regarded as suitable for IVT by European guidelines and experts.

In patients taking direct thrombin inhibitors such as dabigatran, IVT eligibility is routinely determined by assessing thrombin time (IVT eligibility given when thrombin time is < 60 s; outside this limit, application of idarucizumab and IVT are standard) [4]. To our knowledge, determining plasma levels of dabigatran is uncommon.

Our findings demonstrate a high level of concordance with the cut-off values reported by Willekens et al. (2021), who employed the same anti-Xa reagent and used liquid chromatography-tandem mass spectrometry (LC-MS/MS) as the reference method for DOAC quantification [14]. Boissier et al. (2021) reported slightly lower threshold values, which may be attributable to the application of a different statistical method of derivation [13].

Heparin-calibrated anti-Xa assays display some advantages over those determining DOAC plasma levels: (i) they are more widely available, and (ii) they typically employ lower sample dilutions compared to substance-specific calibration assays, reflecting the higher in-vitro sensitivity of these assays to DOACs within their expected pharmacologic range.

Consequently, this methodological difference may confer greater analytical precision, particularly at low DOAC plasma concentrations, which is of clinical relevance when assessing residual anticoagulant activity in the context of acute therapeutic decision-making.

It remains to be determined if a higher anti-factor Xa threshold for IVT may be justified for routine clinical practice. The threshold value of < 0.5 U/ml for heparin-calibrated anti-factor Xa activity proposed by the ESO-Guideline for IVT in AIS means that the DOAC levels for apixaban and rivaroxaban would be below 50 ng/mL. In our cohort, substance specific levels of < 50 ng/ml correlated with factor Xa activity of < 0,55 U/ml for apixaban, < 0,6 U/ml for rivaroxaban and < 0,4 U/ml for edoxaban. Some studies propose an even more liberal approach with IVT-administration in DOAC-levels < 100 ng/ml [7]. In our cohort, substance-specific levels of < 100 ng/ml corresponded to an anti-factor Xa activity < 1,2 for apixaban and rivaroxaban, and < 0,8 U/ml for edoxaban. If an even more liberal approach is warranted in cases with severe neurological impairment (NIH-SS > 6), absence of large vessel occlusion and thus, no possibility of mechanical thrombus evacuation should be determined in future studies including outcome and safety data.

No increased risk of intracranial hemorrhage associated with DOAC use was observed in a large retrospective cohort study on IVT in AIS on 163,038 patients, including 2,207 individuals receiving DOAC therapy, alongside a registry comprising 33,207 patients (225 of whom had available DOAC plasma level measurements and 355 who underwent IVT without prior assessment of DOAC plasma levels or reversal treatment) [10, 15]. In our experience, many DOAC patients with AIS exhibit low or negative anti-Xa activity on admission, which is supported by data from a retrospective study [16]. Furthermore, a small retrospective study reported no difference in the rate of symptomatic intracranial hemorrhage in DOAC patients who received IVT with tenecteplase compared to alteplase [17]. These findings suggest that the presence of DOACs may not independently confer an increased risk of hemorrhagic complications following IVT in the context of AIS.

Limitations of our study include the single-center setting and retrospective design, and the interpretation of the data regarding edoxaban patients warrants particular caution due to their low number. Regarding the latter, this data should be interpreted with caution, particularly in light of the fact that the studies on which the suggestions for critical DOAC-levels (e.g. < 100 ng/ml) are based, mainly included patients with apixaban and rivaroxaban [8]. In turn, it remains unclear, whether a threshold for IVT < 50 or 100 ng/m or < 0,5/< 1,0 U/ml, respectively, is applicable in edoxaban patients. Hence, to determine a universal safe threshold may not be possible, particularly in patients on a DOAC of unknown allocation or with impaired renal function (GFR < 50 ml/min). In our cohort, a significant proportion of patients had an impaired renal function (GFR < 50 or even < 30 ml/min). This represents a limitation of our study and could result in a prolonged DOAC-elimination. In turn, this could increase the bleeding risk and should be considered in future multicenter studies on this topic.

Safety and efficacy of IVT in AIS patients and DOAC intake are currently being prospectively investigated in the randomized and controlled multicenter DO-IT trial (NCT06571149). Another study in the emergency setting found higher DOAC levels in bleeding patients with NVAF than in patients without bleeding symptoms suggesting higher DOAC levels might have an impact on the bleeding risk [18]. Future multicenter studies are necessary to define an ideal threshold at which IVT can be safely performed in AIS patients on DOACs.

## Conclusions

(i) The heparin-calibrated anti-factor Xa activity is suitable for reliably estimating the extent of the DOAC effect.
(ii) A reliable correlation with the substance-specific levels can be deduced: for a substance-specific level of < 50 ng/ml, a standardised threshold value of < 0,55 U/ml (for apixaban) and < 0,6 U/ml (for rivaroxaban) can be derived, for a level of < 100 ng/ml, this changes to < 1,2 U/ml. For edoxaban, this is 0,4 U/ml and 0,8 U/ml, respectively. However, the low number of patients on edoxaban both in our cohort and the studies leading to the guideline recommendations regarding substance specific levels and IVT represent a clear limitation. They should be taken into account when interpreting this data and in particular in patients on DOAC with unknown allocation and/or impaired renal function.
(iii) All threshold values presented are laboratory-derived and require external validation and clinical outcome studies before being used to guide IVT decisions. Future multicentred studies should aim to develop a standardised coagulation diagnostic approach in DOAC-patients with AIS comprising thrombin time and factor Xa activity. This would be particularly useful for hospitals with no access to substance specific level testing.

## Data Availability

No datasets were generated or analysed during the current study.
